# From farm to fork: Microplastic contamination in the meat and dairy supply chain

**DOI:** 10.1016/j.crfs.2026.101334

**Published:** 2026-02-01

**Authors:** Saydur Rahman, Promit Sarker, Tonni Rani Datta, Tasnim Iqbal Maysha, Samiha Rahman, Writam Saha, Aniruddha Sarker, Md. Anisur Rahman Mazumder

**Affiliations:** a Interdisciplinary Institute for Food Security, Bangladesh Agricultural University, Mymensingh, 2202, Bangladesh; b Department of Food Engineering and Technology, Bangladesh Agricultural University, Mymensingh, 2202, Bangladesh

**Keywords:** Microplastics, Meat, Dairy, Food safety, Human health risk

## Abstract

Microplastics (MPs) are now widespread contaminants in both terrestrial and aquatic ecosystems, leading to increasing worries about food safety and public health. This review offers an in-depth evaluation of the prevalence, pathways, and risks associated with MPs in meat and dairy products, which are significant global sources of animal-based nutrition. Data from different countries shows a persistent presence of MP contamination in livestock tissues, poultry organs, processed meat products, raw milk, and commercial dairy items, with identified polymer types such as polyethylene, polypropylene, polystyrene, nylon, PET, and regenerated cellulose. MPs are primarily found in the form of fibers, fragments, films, and irregular particles, with sizes varying from less than 10 μm to several millimeters. Their concentrations can range from a few particles per gram in raw meat to over 30,000 MP/kg in processed products, and from several MPs per liter in raw milk to more than 1800 MP/kg in cheese. Contamination occurs at various points along the farm-to-fork continuum, encompassing ingestion via tainted feed and water, interaction with agricultural plastics, transfer from milking and processing apparatus, wear during cutting and grinding, and leaching from packaging materials. Recent toxicological findings indicate that MPs and their related chemical additives could lead to gastrointestinal inflammation, oxidative stress, endocrine disruption, immunomodulation, and microbiome dysbiosis, although the long-term health effects are still not fully comprehended. Inconsistencies in methodology related to sampling, particle extraction, and spectroscopic identification impede precise comparisons of exposure and assessments of risk. The review points out significant gaps in current studies and emphasizes the necessity for uniform analytical techniques, enhanced waste and plastic management, as well as sustainable processing and packaging approaches to reduce the entry of MPs into animal-derived foods.

## Highlights

• Microplastics found in meat & dairy globally.
• Contamination via feed, packaging, environment.
• Health risks: gut, liver, reproductive harm.
• PE, PS, nylon common in meat/dairy MPs.
• Need better detection methods & standards.

## Graphical abstract

**Figure:**
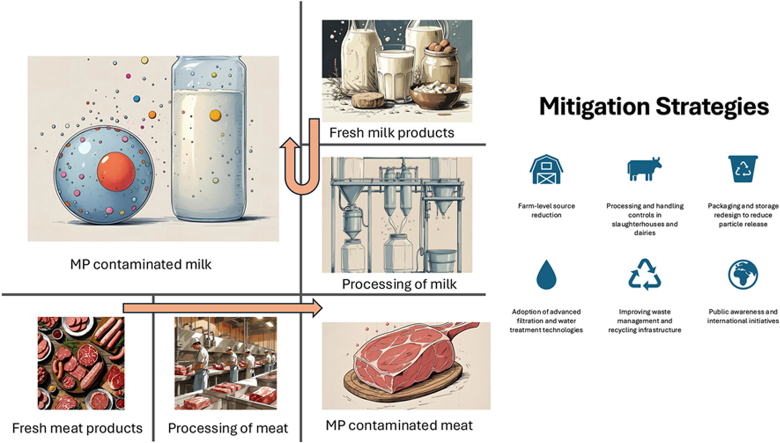

## 1 Introduction

Microplastics (MPs) have become widely acknowledged as widespread environmental pollutants, prompting increasing apprehension regarding their presence in both terrestrial and aquatic ecosystems and their possible infiltration into the food chain (Adjama et al., 2024; Zhang et al., 2022). Due to their persistence, fragmentation, and circulation through air, water, and soil, MPs have become increasingly associated with food safety and human health. This connection has prompted a more detailed investigation into their sources, pathways, and effects within edible systems.

Meat and dairy have attracted significant focus as potential sources of MPs exposure. The worldwide consumption of these products is on the rise, constituting a significant portion of dietary protein and fat intake globally (Guan et al., 2021; Food and Agriculture Organization of the United Nations, 2025). Considering the daily consumption of meat and dairy products by billions, even minimal contamination levels can lead to significant long-term exposure. This highlights the necessity to elucidate the extent and consequences of MPs in these food items.

Meat and dairy products can be accessed by MPs through various interconnected pathways. Livestock can directly consume MPs from contaminated feed, forage, or drinking water, resulting in their transfer into edible tissues or milk (Sheriff et al., 2023). Contamination is influenced by environmental deposition, agricultural inputs, processing environments, and packaging materials, especially with the extensive use of plastics in handling, storage, and transportation (Sheriff et al., 2023; Chinglenthoiba et al., 2025). The various entry points introduce complexities in risk assessment and underscore the necessity of tracking contamination from production through to consumption.

Recent studies have established the occurrence of MPs in different meat and dairy matrices, highlighting the necessity for dependable detection and characterization techniques (Bahrani et al., 2024; Visentin et al., 2024; Binelli et al., 2025; Dileepan et al., 2025). Analytical methods like spectroscopy and microscopy are being utilized more frequently to assess the abundance, size, morphology, and polymer type of MPs. This enhances the precision of evaluating contamination sources and potential control strategies (Chinglenthoiba et al., 2025).

Evidence indicating that dietary intake of MPs and their associated additives may present health risks raises further concerns. Experimental findings suggest potential negative outcomes, such as inflammatory and immune responses, oxidative stress, and intestinal damage, though the degree of these effects in humans is still being actively studied (Jeong et al., 2024; Lee et al., 2023; Sinha et al., 2025). This ambiguity underscores the necessity for a structured integration of existing information.

This review takes a comprehensive approach, moving beyond previous assessments that mainly listed the prevalence of MPs in animal-derived foods. It critically examines the entire supply chain, analyzing contamination pathways from farm-level exposure through to processing, packaging, and storage. Special attention is directed towards (i) the pathways of contamination that are significant to meat and dairy systems, (ii) the toxicological and microbiota-related impacts stemming from the consumption of MPs through meat and dairy products, and (iii) actionable mitigation strategies associated with identifiable critical control points. This review aims to synthesize evidence related to exposure, effects, and mitigation, advancing beyond mere descriptive reporting to establish a framework for interpreting risks and implementing interventions in meat and dairy production systems.

## 2 Literature retrieval methodology

This review presents a narrative critical analysis of the existing literature regarding MP contamination within the meat and dairy supply chain, integrating current evidence on contamination pathways, toxicological effects, and strategies for mitigation. The literature was identified through structured database searches; however, the review does not adhere to a formal PRISMA workflow. However, the vital criteria (inclusion/exclusion, keyword web surfing, and skimming) has been performed to scrutinize the most relevant data archives. In a nutshell, studies were retrieved from Scopus, Web of Science, PubMed, and Google Scholar. The search strategy combined terms related to plastics (“microplastic” OR “nanoplastic”) with meat- and dairy-specific keywords including “meat,” “beef,” “poultry,” “pork,” “lamb,” “dairy,” “milk,” “cheese,” “yogurt,” as well as broader food-exposure terms such as “food chain,” “food contamination,” and “food safety.” Only peer-reviewed articles published in English were included if they reported data on the occurrence, quantification, identification, or toxicological implications of MPs in meat and/or dairy products. Studies that focused exclusively on seafood, bottled water, or plant-based foods, as well as non-peer-reviewed sources (e.g., theses, conference abstracts) and duplicate records, were excluded. For each eligible study, information was extracted on product type, sampling country/region, polymer composition, particle size and morphology, analytical methods, suspected contamination sources, and any reported health or mitigation outcomes. Extracted findings were synthesized to evaluate global contamination patterns, methodological variability, and major research gaps related to MPs in meat and dairy supply chains.

## 3 Occurrence of MPs in meat and dairy products

MP contamination in food has become a significant issue worldwide, with growing evidence suggesting that meat and dairy products are also affected by this phenomenon. Multiple points along animal-derived food chains have shown the presence of MPs, prompting inquiries into potential dietary exposure and the related public health implications (Filho et al., 2021). Contamination levels reported across various studies show significant variability, influenced by factors such as environmental background pollution, livestock production systems, processing practices, and the degree of plastic exposure during handling and packaging (Oyedun and Lawal, 2023; Sharma, 2024).

### 3.1 MPs in meat

Recent studies confirm that MPs occur in a broad range of meat products, indicating that terrestrial food animals can both accumulate and transfer plastic particles into edible tissues. Across livestock, poultry, and processed meats, researchers have identified diverse polymer types, morphologies, and concentrations, pointing to multiple contamination routes throughout production and processing. Table 1 summarizes reported MP prevalence in meat and meat products, showing that contamination is typically lower in raw tissues but can rise substantially after processing and packaging.

**Table 1:** MPs in contaminated meat and meat products.

Product	Polymer	Size	Shape	Color	Concentration	MPs Intake Rate	Method of Detection	Country	Reference
Edible tissues of livestock (cow & sheep meat)	Ny, PS, and LDPE	<25 μm–5000 μm	Fiber, Fragment, Film	Black, White/Transparent, Blue, Red, Yellow/Brown	0.14 to 0.19 MP/items/g (cow) 0.13 MP/items/g (sheep)	0.16 to 0.48 items/kg BW/day	μ-Raman Analysis, SEM–EDS	Iran	Bahrani et al. (2024)
Beef hamburgers (processed patties)	PC, PE, and PP	30.00 and 3154.00 μm	Irregular shapes (95.99%)	gray color (70.16%)	200.00 to 30,300.00 MP/kg	N/A	FTIR	Italy	Visentin et al. (2024)
Quail meat	PE, PVS	1600 (avg. 67.07 ± 29.89 for PE)	Filament, Fragment, Film	Green (PE)	4.80 ± 2.86 (breast), 1.60 ± 1.81 (leg)	0.48 (breast), 0.16 (leg)	Light microscopy, FTIR	Türkiye	Doğan et al. (2024).
Iranian sausages	PE and PS	1–3000 (majority: 1–500)	Fiber (77–89%), Fragment (11–23%)	Black (46%), White/transparent (28%), Blue (14%), Green (7%), Red (4%)	10–175 particles/kg (average: Optical: 25.7 ± 21.68, Fluorescence: 55.45 ± 45.5)	Adults: 804 (optical), 1734 (fluorescence); Children: 3517 (optical), 7589 (fluorescence)	Stereo-/fluorescence microscopy, FTIR, SEM-EDS	Iran	Pirsaheb et al. (2024)
Crop of the farm chicken	PVC (51.2%), LDPE (30.7%), PS (13.6%), and PPH (4.5%).	500–300 μm (63%), 300–150 μm (21%) 150–50 μm (16%)	Fragments (64%), fibres (30%), sheets (3%), foams (2%), and beads (1%),	Red (32%), Yellow (23%), White (12%), Blue (13%), Transparent (6%), and Black (14%),	17.8 ± 12.1 MPs/crop.	N/A	FTIR	Pakistan	Bilal et al. (2023)
Gizzard of the farm chicken		500–300 μm (47%), 150–50 μm (39%), 300–150 μm (14%)	Fragments (53%), fibres (37%), sheets (7%), and foams (3%)	Red (19%), Yellow (32%), White (8%), Blue (9%), Transparent (10%), and Black (22%)	33.25 ± 17.8 MPs/gizzard				
Domestic pig lungs	PA (46.11%)	115.14–1370.43 μm (microscopy) and 20.34–916.36 μm (LDIR)	Fiber	N/A	12 Particles/g(Polarized Microscopy) &180 Particles/g(LDIR)	N/A	polarized light microscopy and LDIR	China	Li et al. (2023)
Fetal pig lungs	PC (32.99%)	115.14–1370.43 μm (microscopy)			6 Particles/g (Polarized Microscopy& 90 Particles/g(LDIR)		polarized light microscopy and LDIR		
Pork	PVC, PP, PE	>700 nm			PVC: 17–690 μg/g; PE: 88–690 μg/g; PP: 63 μg/g	N/A	Pyrolysis-GC/MS	Netherlands	Veen et al. (2022)
Beef	PVC, PS, PE				PVC: 20–26,002 μg/g; PE: 330–7700 μg/g; PS: 77–200 μg/g				
Chicken meat	PE	8.24–1454.5 μm avg.: 104.2 μm	Varied; changes during cooking (e.g., spherical bubbles after grilling).	White, red, yellow, green (matching cutting board colors)	0.03 to 1.19 MP/g	0.03 to 1.19 MP/g	FTIR	United Arab Emirates (UAE) and Kuwait.	Habib et al. (2022)
Cured meat (bacon, mortadella, salami)	LDPE	N/A	N/A	N/A	N/A	N/A	Micro-Raman spectroscopy	Greece	Katsara et al. (2022)

Here, Ny: Nylon, PS: Polystyrene, LDPE: Low-Density Polyethylene, PC: Polycarbonate, PE: Polyethylene, PP: Polypropylene, PVC: Polyvinyl Chloride, PPH: Polypropylene homopolymer, PA: Polyamide, PC: Polycarbonate, PVS: Polyvinyl stearate, SEM-EDS: Scanning Electron Microscopy/Energy Dispersive X-ray Spectrometry, FTIR: Fourier Transform Infrared Spectroscopy, Py-GC/MS: Pyrolysis-Gas Chromatography/Mass Spectrometry, LDIR: Laser Direct Infrared, avg: Average, N/A: Not available.

In raw livestock tissues, Bahrani et al. (2024) detected nylon, polystyrene, and low-density polyethylene (LDPE) in cow and sheep meat, with concentrations of approximately 0.13–0.19 items/g. Fibers and fragments dominated, and particle sizes varied widely (<25–5000 μm), suggesting exposure through contaminated feed, drinking water, and airborne deposition during rearing or slaughter.

Processed meat products appear especially prone to higher MP burdens. Visentin et al. (2024) reported 200–30,300 MP/kg in beef hamburgers, mainly irregular polycarbonate (PC), polyethylene (PE), and polypropylene (PP) particles, implying that grinding, mixing, and repeated plastic-surface contact may amplify contamination. Similarly, Pirsaheb et al. (2024) found 10–175 particles/kg in Iranian sausages, dominated by polyethylene and polystyrene fibers, and their intake estimates suggested greater exposure risk for children than adults. These findings highlight how industrial processing stages may substantially increase MP levels relative to raw meat.

Poultry studies also demonstrate notable MP exposure. In Pakistan, Bilal et al. (2023) identified 17.8 ± 12.1 MPs per crop and 33.25 ± 17.8 MPs per gizzard in farm chickens, with polymers including PVC, LDPE, PS, and PP homopolymer. The presence of sizable fragments and fibers indicates ingestion from contaminated soils, litter, or feed; importantly, consumption of these organs in some cultures represents a direct dietary pathway. Doğan et al. (2024) likewise detected MPs in quail breast and leg meat, reporting PE and PVS particles at levels up to 4.80 ± 2.86 items in breast tissue.

Evidence from internal organs further suggests systemic distribution of MPs in terrestrial animals. Li et al. (2023) observed polyamide (PA) and PC particles in domestic and fetal pig lungs, with concentrations reaching 180 particles/g by LDIR spectroscopy, raising the possibility of placental transfer and early-life exposure. Using pyrolysis-GC/MS, Veen et al. (2022) detected PVC, PE, and PP in pork and beef at microgram-per-gram levels, indicating that mass-based measures may reveal higher plastic loads than particle counts alone. Together, these results underscore that both detection method and meat type strongly influence reported contamination ranges.

Studies conducted from meat products indicate that MPs enter consumable tissues via both biological absorption and mechanical transfer during processing. The notable rise in MP counts in both ground and blended products highlights the ways in which mechanical stress, surface abrasion, and frequent plastic contact contribute to increased contamination levels. This indicates that evaluations of dietary exposure that rely exclusively on raw meat may not accurately reflect actual consumption levels.

### 3.2 MPs in milk and dairy products

A growing number of studies report MP contamination in milk and dairy products, indicating that dairy supply chains are vulnerable to particle entry from farm environments, processing equipment, and packaging materials. Across regions, detected MPs vary in polymer composition, shape, and size, reflecting multiple contamination points along production and distribution. Table 2 summarizes contamination levels across milk and dairy matrices, highlighting that MPs are consistently detected in both raw and commercial products, with higher loads often associated with processing and packaging stages.

**Table 2:** MPs in contaminated milk and dairy products.

Food	Polymer	Size (μm or mm)	Shape	Color	Concentration (per mL or L or kg or %)	MPs intake rate	Method of Detection	Country	References
Cow milk	Cellulose/regenerated cellulose (84%), PE (5%), Acrylic (3%), PS (2%)	350–1000 μm (37%), 1000–2000 μm (26%)	Fibers (smooth/synthetic or irregular/natural)	Blue (32%), black (30%), clear (16%)	1–27 (avg. 3.85) MFs (Microfiber)	6.60 to 35–80 particles/day	FTIR, Microscopy	Italy	Santonicola et al. (2025)
Milk	PSU (57%), PES (26%), PA(17%)	0.5–5 mm	Fiber, Foam, Fragment	Blue, Black, Green, Red, Violet	0.4 MPs/sample	N/A	Stereomicroscopy, FTIR, FESEM-EDS	India	Dileepan et al. (2025)
Cow milk	PE, PET, PP, PAC, PU, PS, ABS, PA, PEG, SI, PVP	0.04–5.77 mm	Fragments, fibers, film, lines, pellets	–	Milk in, multilayer packaging: 35 MPs/3L PET containers: 16 MPs/3L Glass bottles: 7 MPs/3L	1.33 MPs/day	Stereomicroscope, μ-FTIR	Italy	Binelli et al. (2025)
Raw milk (sheep, goats, buffaloes, cows)	PEA, EPC, HNBR, PAM, PARA, CR, PTFE	Average:10–5000 μm 10–20 μm: 0.06% 20–150 μm: 32.03% 150–500 μm: 23.88% 500–1000 μm: 14.70% 1000–5000 μm: 29.32%	Fibre (52.40%), Film, Fragment, Sphere	Black, Blue, Red (most common), Purple, Yellow, Green, Brown, etc	Sheep: 92.73% Goat: 83.64% Buffalo: 92.73% Cow: 89.13% (Overall contamination: 89.28% of samples	0.14–0.22 MP/mL bw/day	SEM-EDS, ATR-FTIR	Turkiye	Zipak et al. (2024)
Cheese	PE, PP, PES, PA, PVF, POM, others (20 types)	24-200+ (one-third 51–100, 20% < 50)	Fragments (77%), Fibers (22%), Beads (<1%)	Multiple (white 60%, gray 30%, black 8%, red 2%)	Fresh Cheese:1280 MP/kg; Ripened Cheese:1857 MP/kg	N/A	μ-FTIR-ATR (high-resolution)	Italy (2025)	Visentin et al. (2025)
Flavored Yogurt	PS, PP, PE, EVA, N6, PC, PU, and others (15 types).	≤50.5 μm (40% particles) 50.5–120 μm (45%) >120 μm (15%) Max size: 1200 μm	Irregular fragments (92%) Spheres (5%) Fibers (3%)	White (60%), Gray (30%), Black (8%), Red (2%)	20% PS, 3% PP, 1% PE of detected particles per Sample; 76% unidentified	0.56 μg/g bw/day (adults)	μ-Raman, SEM	Asia (3 countries)	Ling et al. (2024)
Raw and packaged milk	PE, N66, N6, PET, PVC, PU, PP, SBR, PS, PMMA	N/A	Fibers (mostly)	Black (mostly)	8.33–54 MPs/100 mL	N/A	ATR-FTIR	India	Kundu et al. (2025)
Yogurt and buttermilk	PET, PA (most dominant)	1000–5000 μm	Fibers (mostly)	Transparent (mostly)	Yogurt: 0.63–0.76 MPS/mL Buttermilk: 0.52–0.70 MP/L	N/A	N/A	Iran	Abedi et al. (2025)
Raw milk	PEA	1–10 μm	Fiber	Black, Blue, red	36 MP/200 ml	N/A	ATR-FTIR	Turkiye	Badwanache and Dodamani (2024)
Milk	PMMA, PA, PU, PS, PE	68–2152 μm	Fiber (74%), Fragment (22%), Oval (3%)	Black, Blue, Red, Brown, Gray, Yellow, Golden, Green, Turquoise	12.40 (conventional), 30.86 (organic), 21.25 (raw) MPs/L	0.02–1.06 (adults), 0.09–4.42 (children) MP per kg bw per day	Optical microscopy, μ -FTIR, SEM-EDS, HPLC-FLD, ICP-MS	Romania	Banica et al. (2024)
Powdered milk	PE	>0.1 mm	Fibers	Varied	279.47 particles/kg	N/A	Microscopy, ATR-FTIR	Bangladesh	Chakraborty et al. (2024)
Liquid milk					182.27 particles/L				
Milk powder	N/A	N/A	Fibers, fragments	N/A	10–110 MP/kg	N/A	FTIR	China	Zhang et al. (2023)
Raw Milk	EP	9–4906 μm	Fiber, Fragment	Blue, Black, Red	19 MPs/100 mL	Adults: 259 ± 2; Children/Pregnant women: 173 ± 14 to 346 ± 7 MP/number/d	Optical microscopy, SEM, FTIR	Turkiye	Buyukunal et al. (2023)
Homogenized Milk	PTFE	37–4220 μm	Fiber, Film	Pink, Purple	26 MPs/100 mL				
Pasteurized Milk	PP	50–2503 μm	Fiber, Sphere	Transparent, Red	26 MPs/100 mL				
Ayran (Turkish Dairy Productt)	EP	17–965 μm	Fiber, Film	Blue, Transparent	18 MPs/100 mL				
Milk	PET, PP, PU, EVA, N6	25–5050 μm	Fiber, Fragment	Black, red, green, blue, brown, and gray	6 MP/L	N/A	FTIR	Turkiye	Basaran et al. (2023)
Branded Milk	PE, PP, PA	<500 μm	Fragments, Fibers, Pellets	Blue, Pink, Purple	164–427 MPs/L	35–80MP/Person/day	Microscopy, FTIR	India	Kiruba et al. (2022)
Raw cow's milk	PE, PP, PES, PTFE, PU, PA	≥5 μm (mostly ≤7 μm)	Particles, beads, fibers	N/A	204–625 MPs	N/A	μ-Raman, SEM-EDX	Switzerland	Filho et al. (2021)
Processed liquid cow's milk (whole, skimmed)	PE, PP, PES, PTFE, PS, PU, PSU, PVA		Particles, beads, fibers		172–348 MPs				
Reconstituted powdered cow's milk	PE, PP, PES, PTFE, PA		Particles, beads		356–1004 MPs				

Here, PE: Polyethylene, PS: Polystyrene, PSU: Polysulfone, PES: Polyethersulfone, PA: Polyamide, PET: Polyethylene Terephthalate, PP: Polypropylene, PAC: Polyacrylate, PU: Polyurethane, ABS: Acrylonitrile Butadiene Styrene, PEG=Polyethylene glycol, SI: silicone, PVP: Polyvinylpyrrolidone, PEA: Polyethyl acrylate, EPC: Ethylene-propylene copolymer, HNBR: Hydrogenated nitrile butadiene rubber, PAM: Polyacrylamide, PARA: Polyaramid, CR: Polychloroprene, PTFE polytetrafluoroethylene, N6: Nylon 6, N66: Nylon 66, EVA: Ethylene Vinyl Acetate, PI: Polyisoprene, PVC: Polyvinyl Chloride, PVF: Polyvinylidene fluoride, PES: Polyester Resin, SBS = styrene-butadiene-styrene, SBR: Styrene-butadiene rubber, PC: Polycarbonate, PMMA: Poly(methyl methacrylate), EP: Ethylene propylene, PVA: Polyvinyl Acetate, FTIR: Fourier Transform Infrared Spectroscopy, FESEM: Field Emission Scanning Electron Microscope, EDS: Energy Dispersive X-ray Spectroscopy, ATR-FTIR: Attenuated Total Reflectance-Fourier Transform Infrared Spectroscopy, SEM: Scanning Electron Microscopy, HPLC-FLD: High-Performance Liquid Chromatography Fluorescence Detection, ICP-MS: Inductively Coupled Plasma Mass Spectrometry, N/A: Not available.

Studies on cow milk consistently show fibers as the dominant MP form. In Italy, Santonicola et al. (2025) found polyethylene, acrylic, and polyester, with concentrations ranging from 1 to 27 MP/mL and typical sizes of 350–2000 μm. In India, Dileepan et al. (2025) detected polysulfone, polyethersulfone, and polyamide fibers and fragments in milk (≈0.4 MPs/sample), suggesting that filtration and processing systems may contribute through polymer shedding.

Packaging has emerged as a major driver of MP occurrence in dairy. Binelli et al. (2025) reported that cow milk stored in multilayer packaging contained substantially higher MP loads (35 MPs/3 L) than milk in PET containers (16 MPs/3 L) or glass bottles (7 MPs/3 L). The wide range of polymers detected (e.g., PE, PET, PP, ABS, polyurethane) indicates frequent plastic contact during storage and retail distribution.

Raw milk from multiple animal species also shows widespread contamination. Zipak et al. (2024) detected MPs in 83–93% of raw sheep, goat, buffalo, and cow milk samples in Türkiye, with fiber-like particles predominating (≈52%). The polymer mix, including elastomers, copolymers, and PTFE, points to infrastructural sources such as milking equipment, tubing, gaskets, and storage tanks. Particle sizes ranged broadly (10–5000 μm), implying both macro-scale shedding and finer MP release within farm environments.

Beyond fresh milk, processed dairy products often contain notable MP burdens. Using high-resolution μ-FTIR, Visentin et al. (2025) reported 1280–1857 MP/kg in Italian cheeses, dominated by PE, PP, and PA, with fragments representing most particles. Flavored yogurts analyzed across Asian countries by Ling et al. (2024) contained multiple polymers (PS, PP, PE, EVA, nylon, PC), largely as irregular fragments ≤120 μm, highlighting the contribution of industrial processing and packaged storage.

Several studies also demonstrate variability linked to farming systems and processing context. In Romania, Banica et al. (2024) observed higher MP concentrations in organic milk (30.86 MP/L) than in conventional or raw milk, with fibers (74%) and polymers such as PMMA, PA, PU, PS, and PE. This pattern suggests that packaging, handling, and equipment wear may influence MP levels as strongly as production type itself.

Milk powders represent another exposure route, likely due to high-temperature dehydration and extensive plastic contact during processing. Chakraborty et al. (2024) recorded 279.47 particles/kg in powdered milk in Bangladesh, while Zhang et al. (2023) found 10–110 MP/kg in Chinese milk powder, with fibers and fragments frequently observed.

The literature indicates consistent MP presence in milk and dairy products, but direct comparison across studies remains difficult because detection methods and reporting units differ (e.g., particles per mL, L, or kg). Standardized protocols will be essential for refining global exposure estimates and identifying the most critical control points in dairy supply chains.

Milk and dairy products appear especially susceptible to MP contamination because of their significant interaction with plastic materials throughout the processes of milking, processing, and packaging. The prevalence of fibers indicates a consistent, low-level release from machinery and packaging, rather than random contamination incidents. Variations noted among packaging types indicate the selection of materials can significantly affect consumer exposure. The findings underscore the importance of focusing on dairy systems for advancements in packaging innovation and the redesign of equipment to minimize MP release.

## 4 Contamination pathways of MPs in meat and dairy products

**Fig. 1:**
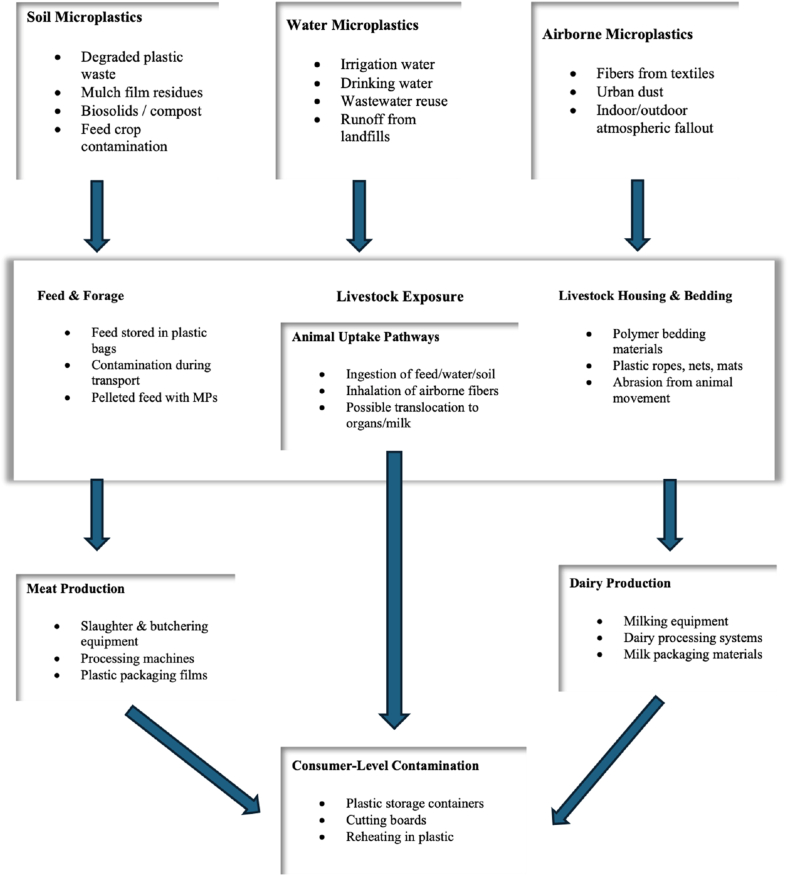
Farm-to-fork pathways of MP contamination in meat and dairy.

MP contamination in meat and dairy products originates from various stages within the farm-to-fork continuum. Plastics are extensively utilized in agricultural production, animal husbandry, food processing, and packaging. Consequently, MPs may infiltrate animal-derived foods via environmental exposure, equipment degradation, packaging interactions, and post-processing handling. The pathways frequently intersect, resulting in contamination as a cumulative process rather than an isolated occurrence. Fig. 1 depicts the pathways of MP contamination in meat and dairy from farm to fork.

### 4.1 Feed- and water-mediated contamination

Livestock exposure to MPs can occur through contaminated feed and drinking water, reflecting the pervasive presence of MPs in terrestrial and aquatic environments. Feed materials may contain MPs originating from agricultural soils amended with plastic mulches, compost, manure, or biosolids, as well as from airborne deposition during harvesting, transport, and storage (Nourozi et al., 2024). Drinking water represents an additional pathway, particularly where surface water or insufficiently filtered supplies are used (Khan et al., 2024). Upon consumption, particles can persist in the gastrointestinal tract or possibly migrate to consumable tissues and mammary glands, facilitating their incorporation into meat and milk (Sheriff et al., 2023). Feed that is stored or transported in plastic sacks and containers has the potential to accumulate additional particles prior to reaching animals, thereby heightening exposure levels. Although ingestion through feed and water likely represents a chronic, low-level exposure, it provides a continuous input route that may contribute to MP presence in edible tissues and milk, depending on particle size, polymer type, and translocation potential.

### 4.2 Environmental and airborne exposure

Airborne MPs represent a frequently neglected route of contamination in livestock housing, milking parlors, and processing environments. Suspended MPs in indoor and outdoor air may derive from deteriorated building materials, synthetic textiles, packaging, and proximate industrial or urban sources (Khan et al., 2024). These particles may accumulate on feed, water, animal surfaces, and exposed meat or dairy products (Zuri et al., 2023). Airborne MPs, being typically smaller, may significantly contribute fine particles that are challenging to detect and control (Torres-Agullo et al., 2021). This underscores the importance of ventilation, hygiene, and environmental background contamination in food production systems.

### 4.3 Processing and handling-related contamination

Post-harvest processing represents one of the most significant amplification points for MP contamination. During processing, direct contact between food products and plastic tools or machinery can introduce MPs through abrasion, friction, and thermal degradation. Cutting boards, conveyor belts, storage bins, and processing surfaces made of polyethylene or polypropylene frequently shed particles during routine operations (Sharma, 2024; Habib et al., 2022; Chinglenthoiba et al., 2025). Each processing step adds cumulative contact events, increasing the likelihood of MP release into meat and dairy matrices. Unlike farm-level exposure, these pathways are highly process-dependent and therefore more amenable to targeted control through equipment selection, maintenance, and process optimization.

### 4.4 Packaging- and storage-related contamination

Packaging materials are another significant source of contamination, particularly during prolonged storage. Meat and dairy products stored in plastic films, multilayer packaging, or disposable containers may accumulate MPs due to mechanical stress, temperature fluctuations, or UV exposure during distribution and storage (Kedzierski et al., 2020). Studies showing higher MP levels in products packaged in multilayer plastics compared to PET or glass highlight the influence of packaging composition on contamination levels (Binelli et al., 2025). The elevated levels of MPs found in foods packaged in multilayer plastics can be attributed to the intricate structure of these materials, which consist of various polymer layers adhered together by adhesives. Mechanical stress encountered during handling, sealing, transport, and storage may lead to interlayer delamination and polymer abrasion, which in turn may increase particle release. Packaging often serves as the final point of contact prior to consumption, and the MPs introduced at this stage can significantly affect consumer exposure, emphasizing the crucial significance of packaging design and storage conditions.

Contamination of meat and dairy products by MPs typically does not originate from a singular predominant pathway. It reflects the aggregate contribution of various sources operating throughout the production chain, encompassing environmental exposure at the farm level as well as inputs related to processing and packaging. Interactions between pathways, including airborne deposition onto packaging materials and recontamination during handling, complicate source attribution. The cumulative aspect emphasizes the necessity for cohesive mitigation strategies and uniform monitoring methods to pinpoint essential control points.

## 5 Health implications of MP ingestion

Recent studies have not provided definitive insights into the effects of MPs on human health; nonetheless, new toxicological and epidemiological findings underscore various potential risks linked to dietary exposure. The detection of MPs in various meat and dairy products has raised significant concerns regarding the potential cumulative physiological effects of continuous ingestion, even at low levels.

Recent intake assessments highlight the significance of this issue. The estimated intake of MPs from meat and dairy products shows significant variation depending on the specific products and geographical locations, highlighting the impact of differing production systems, processing methods, and packaging practices. Intake levels for meat products vary between 0.16 and 0.48 MP/kg body weight/day from livestock tissues (Bahrani et al., 2024). In contrast, heavily processed items like sausages lead to significantly higher exposures, ranging from 804 to 1734 MPs/day for adults and 3517–7589 MPs/day for children (Pirsaheb et al., 2024). The observed difference can probably be attributed to the accumulation of contamination throughout various processing stages, including grinding, mixing, and emulsification. These stages increase friction and interaction with polymer-based equipment, in addition to prolonged exposure to plastic packaging and storage. Collectively, these elements offer credible pathways for the significantly elevated MP levels detected in processed meat products.

Poultry products can be important contributors, with chicken meat containing up to 1.19 MP/g depending on the cooking method and cutting surfaces (Habib et al., 2022). Dairy products serve as a consistent source of exposure, with cow milk providing between 6.6 and 35–80 MPs per day, varying by brand and packaging type (Santonicola et al., 2025; Kiruba et al., 2022). Additionally, Romanian milk products contribute 0.02–1.06 MPs per kg body weight per day for adults and 0.09–4.42 MPs per kg body weight per day for children (Banica et al., 2024). The consumption of processed dairy products, including flavored yogurt, contributes to an increased potential exposure, quantified at 0.56 μg/kg bw/day (Ling et al., 2024). The findings suggest that the daily intake of animal-derived foods could be a significant and consistent source of MP consumption. This accumulating evidence highlights the necessity of comprehending the interactions between ingested MPs and the human body, as well as the potential risks they may present.

### 5.1 Physical effects of MPs

MPs exert significant physical effects on biological systems primarily due to their small size, irregular shapes, and persistence. When ingested or inhaled, MPs can build up in various tissues, especially within the gastrointestinal tract and respiratory system, potentially leading to mechanical irritation, abrasion, and obstruction (Nawab et al., 2024). In the gastrointestinal tract, it has been observed that MPs adhere to the mucosal surface, disrupt epithelial integrity, and increase intestinal permeability, a phenomenon often referred to as “leaky gut.” This physical disruption enables the movement of MPs and related contaminants through epithelial barriers into systemic circulation, consequently enhancing downstream health effects (Abbas et al., 2025).

In addition to the gastrointestinal tract, MPs and related nanoscale particles have the potential to migrate into various tissues, potentially leading to oxidative stress and dysfunction of organs. Findings from animal and in vitro studies indicate effects on the lungs, liver, kidneys, heart, immune system, and nervous system (Das, 2023). Reproductive toxicity is an increasingly important issue; MPs have been associated with disruptions in the blood–testis barrier, impaired spermatogenesis, ovarian atrophy, placental dysfunction, and altered fetal development (Wang et al., 2024; Anifowoshe et al., 2025). Increased exposure to PET MPs has also been demonstrated to lower sperm count and harm the structure of the epididymis (Jeong et al., 2025). The collective findings underscore a wide range of possible physical effects, yet their significance concerning human dietary exposures necessitates additional research.

### 5.2 Toxicological effects of MPs

Beyond physical stress, MPs induce a broad range of toxicological effects mediated by oxidative stress, inflammation, and chemical toxicity. MPs can directly stimulate the production of reactive oxygen species (ROS), leading to oxidative damage of lipids, proteins, and DNA (Kadac-Czapska et al., 2024). This oxidative stress activates pro-inflammatory signaling pathways, including NF-κB and inflammasome cascades, resulting in the sustained release of cytokines such as IL-1β, IL-6, and TNF-α. Chronic activation of these pathways has been linked to tissue injury, immune dysregulation, and the progression of non-communicable diseases (Abbas et al., 2025).

In addition, MPs act as vectors for toxic chemicals, including plastic additives and adsorbed environmental pollutants. The intricate nature of polymer matrices allows for the potential release of detrimental compounds, including phthalates, bisphenol A (BPA), polycyclic aromatic hydrocarbons (PAHs), and polychlorinated biphenyls (PCBs), all of which are recognized as endocrine disruptors or carcinogenic substances. Phthalates, frequently utilized as plasticizers, have been linked to compromised thyroid function (Bereketoglu and Pradhan, 2022) and negative impacts on ovarian health (Neff and Flaws, 2021).

BPA raises significant concerns due to its potential to disrupt hormone signaling pathways, leading to disturbances in the hypothalamus–pituitary–gonadal and adrenal axes (Tarafdar et al., 2022). It might also inhibit immune function through the downregulation of T-cell activity (Park et al., 2022). In a similar vein, MPs associated with PAHs have been connected to lung cancer (Stading et al., 2021) as well as cardiovascular diseases, such as hypertension and myocardial infarction (Mallah et al., 2021). Polystyrene MPs have been associated with cellular apoptosis, mitochondrial dysfunction, and altered metabolic signaling in experimental models (Tang et al., 2024; Abbas et al., 2025). The buildup of MPs could potentially play a role in the development of systemic diseases. Recent investigations link the presence of MPs to the advancement of atherosclerosis (Yu et al., 2024), the formation of gallstones (Zhang et al., 2024), neurodegenerative conditions like Alzheimer's and Parkinson's disease (Gou et al., 2024), as well as metabolic disorders such as nonalcoholic fatty liver disease (Shi et al., 2025). The combined physical presence of MPs and their chemical payload create a synergistic toxicological burden that heightens health risks.

### 5.3 Effects of MPs on microbiota

MPs have the potential to influence human health by altering the gut microbiome. Experimental studies indicate that exposure to MPs can lead to intestinal dysbiosis, characterized by an increase in the abundance of bacteria such as *Firmicutes*, *Proteobacteria*, and *Chlamydia*, while simultaneously decreasing beneficial groups like Bacteroidetes (Souza-Silva et al., 2022). MP exposure promotes the proliferation of pro-inflammatory and opportunistic bacteria, including *Escherichia*–*Shigella*, *Bilophila*, *Streptococcus*, *Treponema*, and *Porphyromonas*, while suppressing health-associated genera such as *Lactobacillus*, *Parabacteroides*, *Alistipes*, and *Blautia* (Thin et al., 2025). This imbalance could lead to metabolic dysfunction, inflammation, and compromised gastrointestinal health.

Exposure to MPs is linked to a decline in microbial diversity, as indicated by reductions in α-diversity indices across various polymer types. A decrease in microbial diversity is acknowledged as an indicator of gut dysbiosis and has been associated with a heightened risk of chronic intestinal and metabolic diseases (Thin et al., 2025). MPs interfere with microbial metabolic pathways, especially the production of short-chain fatty acids (SCFAs) (Bora et al., 2024). Lower levels of acetate and butyrate compromise gut barrier integrity and immune regulation, while changes in carbohydrate, lipid, and amino acid metabolism additionally lead to metabolic imbalance (Thin et al., 2025).

MPs could also engage with microbial communities in manners that increase toxicity. Exposure to PET MPs has been demonstrated to modify the functional profile of human colonic microbiota (Tamargo et al., 2022). Certain gut bacteria can enhance the release of phthalates and other plastic additives from MPs, thereby elevating the presence of these detrimental chemicals in the intestinal environment, while also suppressing normal microbial metabolism (Yan et al., 2022). The interactions among microorganisms suggest that MPs could intensify inflammatory responses, compromise the integrity of the intestinal barrier, and lead to more extensive systemic health implications.

While the direct causal relationships between dietary MP exposure and human health are still being explored, estimates of intake from meat and dairy products indicate a pattern of chronic and repeated exposure. Certain populations, especially children, may encounter significantly elevated risks because of body weight–adjusted intake and consumption patterns. The interplay of physical particle effects, chemical toxicity, and microbiome disruption indicates that MPs could function as multifactorial stressors instead of singular hazards, highlighting the importance of precautionary risk management.

## 6 Mitigation strategies for MPs in meat and dairy products

**Fig. 2:**
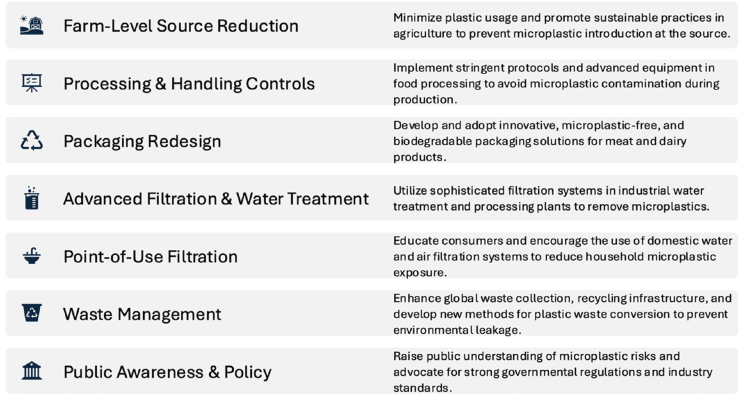
Mitigation strategies of MP in meat and dairy products.

Addressing MP contamination in meat and dairy requires a comprehensive approach, as particles can infiltrate at various stages: from the farm, during transportation, throughout processing, and through packaging and consumer interactions. To achieve optimal outcomes, it is essential to integrate source reduction, engineering controls, good manufacturing practices, packaging redesign, and monitoring, all supported by policy and awareness initiatives. Fig. 2 illustrates the strategies employed to mitigate MPs contamination in meat and dairy products.

### 6.1 Farm-level source reduction (feed, water, housing, and agricultural plastics)

Because livestock can ingest MPs through contaminated water, feed, and airborne deposition (Khan et al., 2024), it is essential to focus on cleaner inputs and minimizing plastic degradation on farms as a priority for mitigation efforts. Actions involve reducing fragmentation from silage films, twines, bedding materials, and plastic storage sacks; enhancing the handling and storage of feed to minimize contact abrasion; and avoiding the introduction of MPs through manure, compost, biosolids, or wastewater-irrigated forage where applicable (Mohasin et al., 2025). The implementation of biodegradable or more resilient agricultural films, enhanced collection of damaged plastics, and optimized management of manure and biosolids can effectively reduce the accumulation of MPs in soils and feed-crop pathways.

### 6.2 Processing and handling controls in slaughterhouses and dairies

Processing environments have the potential to increase MP loads due to continuous interaction with plastic surfaces such as conveyor belts, cutting boards, bins, tubing, and gaskets, as well as through mechanical wear that occurs during cutting, grinding, and mixing (Melikoglu, 2025). Efforts should concentrate on areas experiencing significant wear, implementing proactive maintenance through the timely replacement of degraded polymer components, refining cleaning procedures to prevent increased abrasion, and reengineering workflows to minimize unnecessary interactions with plastic. When possible, transitioning certain high-friction components to materials that are more resistant to abrasion could lead to a decrease in MP generation. However, this should be confirmed through site-specific monitoring and standardized testing.

### 6.3 Packaging and storage redesign to reduce particle release

Packaging consistently plays a significant role in the presence of MPs in dairy and processed meat, especially in situations involving multilayer films or where there is frequent mechanical stress and temperature variations. Mitigation involves minimizing the use of multilayer plastics when they are not essential, enhancing packaging integrity to prevent cracking and shedding, and selecting packaging formats that are less likely to release particles. It is important to acknowledge that performance is influenced by factors such as polymer type, additives, aging, UV exposure, and mechanical stress (Chinglenthoiba et al., 2025). It is essential to provide storage guidance that advises against extended exposure to heat and sunlight, as these factors can hasten material degradation and lead to particle release.

### 6.4 Adoption of advanced filtration and water treatment technologies

Water serves as a significant pathway for the transport and introduction of MPs into the meat and dairy supply chain, making the implementation of advanced filtration and water treatment technologies a crucial mitigation strategy. Treatment methods including membrane filtration, sand filtration, and nanofiltration have shown efficacy in decreasing MP concentrations across various particle sizes, thus restricting their infiltration into livestock drinking water, irrigation sources for feed production, and processing water systems (Gao et al., 2022; Bodzek and Bodzek, 2025).

At the municipal level, enhancing drinking water and wastewater treatment processes can mitigate the environmental release and recirculation of MPs, thereby indirectly reducing the background contamination that reaches agricultural systems. Implementing point-of-use filtration in drinking water lines, milking systems, and processing facilities at both farm and industrial levels can serve as an effective additional barrier against the introduction of MPs, especially in areas dependent on surface water or recycled water sources. The expenses and operational complexities of these technologies differ, yet their incorporation into water management practices provides a viable approach to decreasing MP inputs throughout various phases of meat and dairy production. This approach works in tandem with source-control and material-based mitigation strategies.

### 6.5 Improving waste management and recycling infrastructure

Efficient management of plastic waste is crucial for reducing environmental MP levels. Improperly handled plastic waste breaks down into MPs, leading to contamination of soil, water, and air, which eventually affects livestock feed and dairy settings. Enhancing waste collection, optimizing recycling systems, and investing in circular plastic economies can lead to a substantial decrease in environmental contamination (Suzuki et al., 2024; Samitthiwetcharong et al., 2024). Infrastructure development and policy support spearheaded by the government are essential for mitigating plastic leakage into agricultural ecosystems.

### 6.6 Public awareness and international initiatives

Informing farmers, food processors, policymakers, and consumers about the origins and dangers of MPs is essential for effective long-term solutions. Awareness campaigns can enhance responsible plastic use, encourage proper disposal practices, and foster informed purchasing decisions, such as selecting products with sustainable packaging or backing plastic-free farming initiatives. Public awareness campaigns, like the “Plastic Free July” initiative, have shown significant potential worldwide in decreasing reliance on single use plastics (Plastic Free Foundation, 2022). A heightened awareness among the public can catalyze transformations within the industry and bolster regulatory initiatives focused on mitigating MP pollution across the food supply chain. Economic policies, including tax incentives and Extended Producer Responsibility (EPR), drive the transition to sustainable packaging by shifting waste management accountability to producers. Financial mechanisms, such as the UK Plastic Packaging Tax, effectively catalyze industrial shifts by penalizing low recycled content and incentivizing eco-design innovation (Rahman et al., 2025a).

The outlined mitigation strategies demonstrate that it is technically achievable to reduce MP contamination, though it necessitates collaborative efforts throughout the supply chain. Immediate benefits can be achieved through source reduction and processing controls, whereas packaging redesign and enhanced waste management present solutions that are more sustainable in the long run. It is crucial that mitigation efforts focus on interventions aimed at reducing contamination while maintaining food safety, quality, and accessibility, thereby ensuring that sustainability goals are in harmony with public health goals.

## 7 Commonly used analytical methods in MPs assessment

**Fig. 3:**
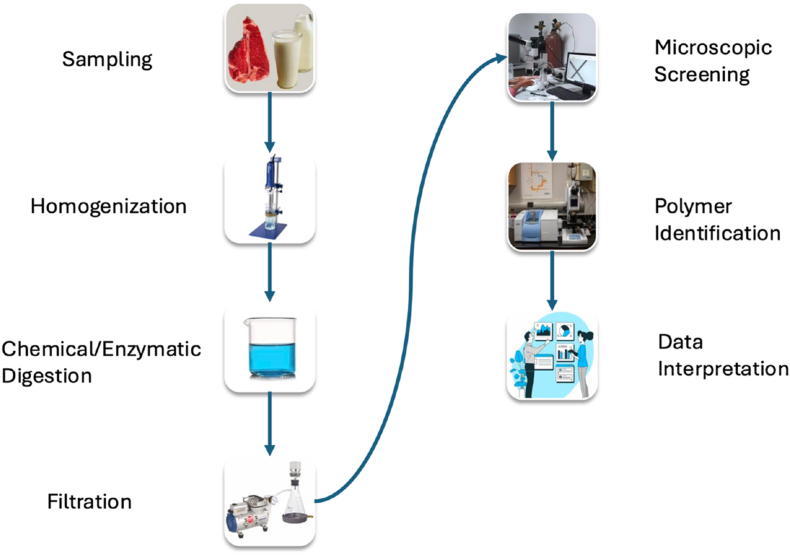
Analytical workflow for MP detection in meat and dairy.

Accurate detection and characterization of MPs in food matrices particularly meat and dairy products require analytical techniques capable of distinguishing particles by size, morphology, and polymer type. However, food systems pose significant challenges due to their complex organic composition, which often necessitates rigorous sample preparation and specialized instrumentation. The following methods represent the most widely applied analytical approaches, each offering distinct advantages and limitations depending on the goals of analysis. Implementing validated protocols and utilizing advanced techniques like FTIR, Raman spectroscopy, and pyrolysis-GC/MS will enhance regulatory decision-making and streamline routine surveillance programs (Adjama et al., 2024; Fiore et al., 2023; Rahman et al., 2025b). Fig. 3 presents analytical workflow for MP detection in meat and dairy.

### 7.1 Microscopy-based techniques

Microscopy provides the first line of assessment for identifying and quantifying MPs based on visible characteristics such as size, shape, and color. Stereomicroscopy is commonly used for preliminary screening because it is cost-effective and suitable for larger particles (Makhdoumi et al., 2021). Optical microscopy allows closer visualization of particle morphology, though its resolution is insufficient for detecting very small MPs (Shruti and Kutralam-Muniasamy, 2023).

Scanning Electron Microscopy (SEM) offers substantially higher resolution and allows detailed examination of MP surface structure, making it valuable for detecting small or irregular particles within complex matrices (Brochado et al., 2024; Akbulut et al., 2024). Field Emission SEM (FESEM) provides even greater detail and is often used when fine structural characterization is required (Wang et al., 2023). While these techniques provide essential morphological information, they cannot independently confirm polymer identity.

### 7.2 Spectroscopic techniques

Spectroscopy continues to be fundamental in the identification of polymers. Fourier Transform Infrared (FTIR) spectroscopy serves as a crucial method for identifying chemical composition through the comparison of particle spectra with established reference libraries. This method can distinguish between various polymer types and is applicable in techniques like μ-FTIR and ATR-FTIR for analyzing smaller particles or irregular samples (Brochado et al., 2024; Silva et al., 2024; Akbulut et al., 2024).

Raman spectroscopy provides additional insights by identifying molecular vibrational signatures with exceptional spatial resolution. Micro-Raman proves to be particularly valuable for the characterization of particles smaller than 20 μm and can identify polymers that may be challenging to differentiate using FTIR alone (Shruti and Kutralam-Muniasamy, 2023; Espiritu et al., 2024; Du et al., 2024). Both FTIR and Raman spectroscopy necessitate clean, isolated particles and may be influenced by food residues or fluorescence.

### 7.3 Mass spectrometry-based techniques

Pyrolysis–Gas Chromatography–Mass Spectrometry (Py-GC-MS) is an advanced technique that thermally breaks down MPs into identifiable fragments, allowing for both quantitative and qualitative evaluation of polymer types. This approach proves to be especially beneficial for intricate mixtures or in cases where particles are either too diminutive or degraded to allow for spectroscopic identification (Velimirovic et al., 2020; Wang et al., 2023). Recent evaluations reveal challenges including high detection limits and interference from food matrices, emphasizing the necessity for careful sample preparation (Jeffries et al., 2025).

### 7.4 Other emerging techniques

Single-Particle Inductively Coupled Plasma Mass Spectrometry (spICP-MS) is an emerging approach capable of detecting particle size and number concentration, originally designed for nanomaterials. While still in its early stages for MP testing, it shows promise for polymers containing metal additives or pigments, enhancing trace-level detection and characterization (Velimirovic et al., 2020).

Analytical limitations remain a significant challenge to precise exposure assessment. Differences in detection thresholds, polymer identification, and reporting units impede comparability and regulatory interpretation. Enhancing standardized and validated techniques, especially for smaller MPs and nanoplastics, will be crucial for converting scientific discoveries into actionable food safety regulations and monitoring initiatives.

## 8 Research gaps and future directions

Despite the increasing focus on MP contamination in food systems, notable gaps remain, especially concerning meat and dairy products. The absence of standardized methods for sampling, identifying, and quantifying MPs within complex food matrices presents a significant limitation (Wang et al., 2023). Discrepancies in reported findings stem from variations in current methodologies concerning identification techniques (e.g., FTIR, Raman spectroscopy), filtration systems, and digestion protocols. Precise evaluation of risks and regulatory measures relies on the development of universally recognized protocols for analyzing MPs in animal-derived food products.

Investigating the bioaccumulation mechanisms of MPs and their fate within animal organisms represents a significant gap in current knowledge (Jeong et al., 2024). The movement, durability, and possible transformation of MPs within various tissues particularly in edible components such as muscle and organs require thorough examination. The impact of long-term dietary exposure to MPs on human health remains largely uncharted (Li et al., 2024). To evaluate the effects of MPs and the chemical additives or adsorbed pollutants they carry on human physiology, metabolism, and the microbiome, there is a critical need for toxicological research.

There remains an insufficient body of work examining the impact of agricultural methods on MP contamination, whether they contribute to or mitigate it. The impact of plastic utilization in livestock management, including bedding materials, feeding equipment, and silage films, on contamination levels remains inadequately explored. Future studies should explore the potential of sustainable agricultural innovations, including circular farming systems and biodegradable substitutes, to mitigate MP contamination in the food chain. There is a significant lack of data from low- and middle-income countries. There is a pressing requirement for localized studies because of the variations in food safety regulations and plastic waste management systems among various nations, particularly in areas where smallholder farming and informal markets prevail. This data is essential for both global risk frameworks and the development of locally relevant mitigation strategies.

Future studies should priorities interdisciplinary methods that combine environmental science, food technology, toxicology, and public health. Enhancements in the detection of MPs could be achieved through the application of molecular techniques, the integration of machine learning in spectral analysis, and the utilization of advanced sensor technologies. To evaluate consumer behavior, public perception, and the financial implications of MP contamination and control strategies, socioeconomic studies play a vital role.

## 9 Conclusion

MP contamination in meat and dairy products is evident in global food systems, arising from environmental exposure, agricultural practices, processing equipment, and packaging. The extensive variety of identified polymers and particle types highlights the susceptibility of the entire farm-to-fork continuum. Emerging studies suggest potential health risks such as inflammation, oxidative stress, endocrine disruption, and microbiome imbalance; however, the long-term effects of dietary MP exposure remain uncertain. Inconsistent analytical methods impede progress by restricting reliable comparisons of contamination levels. To protect public health, it is essential to reduce plastic use, improve waste management, enhance filtration technologies, and develop standardized detection protocols. Enhancing interdisciplinary research and increasing data collection, particularly from underrepresented regions, is essential for effective risk assessment and mitigation.

## CRediT authorship contribution statement

**Saydur Rahman:** Conceptualization, Data curation, Investigation, Visualization, Writing – original draft, Writing – review & editing, Supervision. **Promit Sarker:** Writing – original draft, Data curation. **Tonni Rani Datta:** Writing – original draft, Data curation. **Tasnim Iqbal Maysha:** Writing – original draft, Data curation. **Samiha Rahman:** Writing – original draft, Data curation. **Writam Saha:** Writing – original draft, Investigation, Data curation. **Aniruddha Sarker:** Writing – original draft. **Md. Anisur Rahman Mazumder:** Writing – review & editing.

## Author statement

The author affirms that this manuscript, titled “**From Farm to Fork: Microplastic Contamination in the Meat and Dairy Supply Chain**,” represents original research that has not been published before and is not currently being considered for publication elsewhere. All utilized information sources have been duly cited. The individual has exclusively engaged in the conception, design, data collection, analysis, and writing of the manuscript. The author has no conflicts of interest to disclose concerning this work. Revisions were implemented based on feedback from editorial reviews, and the current version represents the author's diligent effort to thoroughly address all comments and enhance the clarity and quality of the manuscript.

## Declaration of competing interest

The authors declare that they have no known competing financial interests or personal relationships that could have appeared to influence the work reported in this paper.

## Data Availability

Not applicable.
